# Synthesis and crystal structures of 3,6-di­hydroxy­picolinic acid and its labile inter­mediate dipotassium 3-hy­droxy-6-(sulfonato­oxy)pyridine-2-carboxyl­ate monohydrate

**DOI:** 10.1107/S2056989021004904

**Published:** 2021-05-14

**Authors:** Edward J. Behrman, Sean Parkin

**Affiliations:** a Department of Chemistry & Biochemistry, The Ohio State University, 484 W. 12th Avenue, Columbus, Ohio, 43210, USA; b Department of Chemistry, University of Kentucky, 505 Rose Street, Lexington, Kentucky, 40506, USA

**Keywords:** Elbs oxidation, crystal structure, space group *Abm*2, hydrogen bonding, inversion twinning, disorder

## Abstract

A simplified two-step synthesis of 3,6-di­hydroxy­picolinic acid (3-hy­droxy-6-oxo-1,6-di­hydro­pyridine-2-carb­oxy­lic acid), C_6_H_5_NO_4_ (**II**), an inter­mediate in the metabolism of picolinic acid, is described. The crystal structure of **II**, along with that of a labile inter­mediate, dipotassium 3-hy­droxy-6-(sulfonato­oxy)pyridine-2-carboxyl­ate monohydrate, 2K^+^·C_6_H_3_NO_7_S^2−^·H_2_O (**I**), is also described. Compound **I** comprises a pyridine ring with carboxyl­ate, hydroxyl (connected by an intra­molecular O—H⋯O hydrogen bond), and sulfate groups at the 2-, 3-, and 6-positions, respectively, along with two potassium cations for charge balance and one water mol­ecule of crystallization. These components are connected into a three-dimensional network by O—H⋯O hydrogen bonds arising from the water mol­ecule, C—H⋯O inter­actions and π–π stacking of pyridine rings. In **II**, the ring nitro­gen atom is protonated, with charge balance provided by the carboxyl­ate group (*i.e*., a zwitterion). The intra­molecular O—H⋯O hydrogen bond observed in **I** is preserved in **II**. Crystals of **II** have unusual space-group symmetry of type *Abm*2 in which extended planar networks of O—H⋯O and N—H⋯O hydrogen-bonded mol­ecules form sheets lying parallel to the *ac* plane, constrained to *b* = 0.25 (and 0.75). The structure was refined as a 50:50 inversion twin. A minor disorder component was modeled by reflection of the major component across a mirror plane perpendicular to *c*.

## Abstract 2

A two-step synthesis of 3,6-di­hydroxy­picolinic acid, its crystal structure and that of a labile inter­mediate, are described.

## Chemical context   

3,6-Di­hydroxy­picolinic acid (3-hy­droxy-6-pyridone-2-carb­oxy­lic acid), C_6_H_5_NO_4_, is an inter­mediate in the metabolism of picolinic acid by several microorganisms (Shukla & Kaul, 1973 ▸; Shukla *et al.*, 1977 ▸; Qiu *et al.*, 2019 ▸). It was isolated from culture media and partially characterized by Shukla & Kaul (1973 ▸; Shukla *et al.*, 1977 ▸) whose work is misrepresented by Qiu *et al.* (2019 ▸) by stating that their work was only theoret­ical. It was synthesized by a six-step procedure from 3-hy­droxy­picolinic acid and characterized by mass spectrometry and NMR data (Qiu *et al.*, 2019 ▸, with C. Shen).

**Figure 1:**
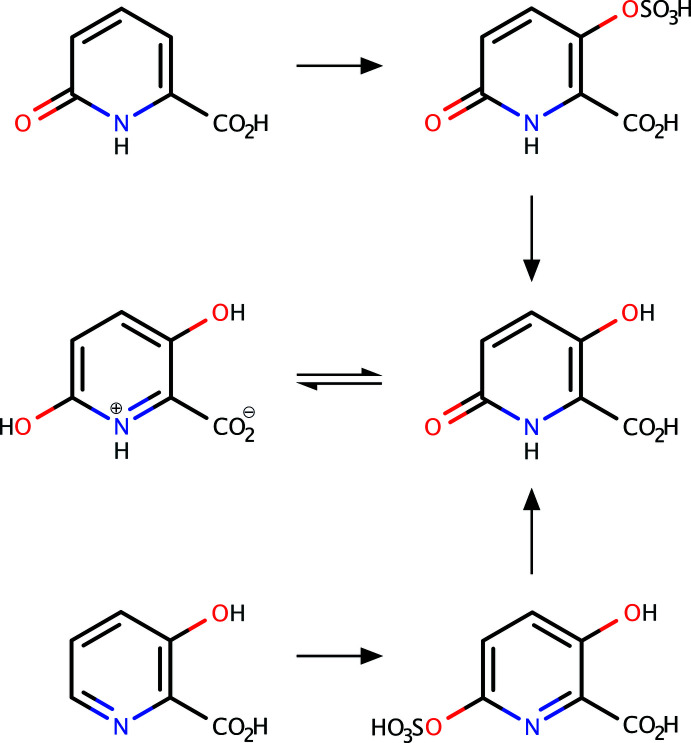
Two synthetic routes to 3,6-di­hydroxy­picolinic acid (**II**), showing tautomerism of the product.

We report here a two-step synthesis also starting with 3-hy­droxy­picolinic acid by an Elbs oxidation (Behrman, 1988 ▸; 2021 ▸) and crystal structures of both the inter­mediate sulfate ester (**I**) and of 3,6-di­hydroxy­picolinic acid (**II**). We considered two routes, as shown in Fig. 1 ▸. Both give the desired product but we chose the pathway from 3-hy­droxy­picolinic acid because in the first step, the dipotassium salt of the 6-sulfate ester precipitates from the mixture in an almost pure state.
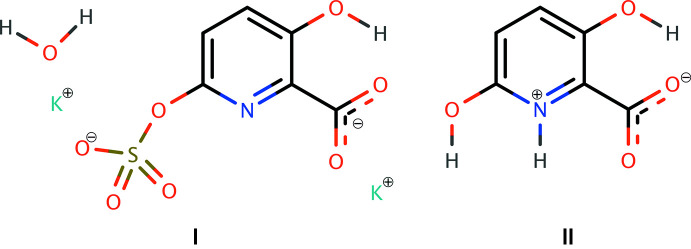


**Figure 2:**
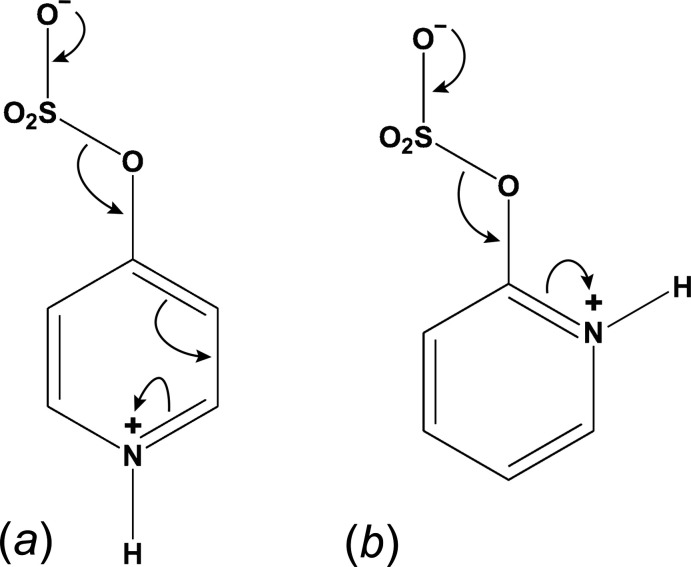
(*a*) A reasonable representation for the mechanism of hydrolysis for the 4-sulfate. (*b*) The *o*-sulfate analog ought to behave similarly.

This sulfate ester is extraordinarily sensitive to acid-catal­yzed hydrolysis; acidification at room temperature (RT) gives complete hydrolysis in a few minutes. The isomeric 6-oxo-picolinic acid-3-sulfate is much more stable although subject to anchimeric assistance (Benkovic, 1966 ▸); it is not completely hydrolyzed after 22 h, RT, pH 2. Nantka-Namirski & Rykowski (1972*a* ▸,*b* ▸) used boiling 20% sulfuric acid for four hours to effect the hydrolyses of the 5-sulfate esters of two di­hydroxy­nicotinic acids. For the rapid hydrolysis of pyridyl-4-sulfate see Jerfy & Roy (1970 ▸) and Goren & Kochansky (1973 ▸) for the effects of impurities. A reasonable representation of the mechanism of the hydrolysis for the 4-sulfate is shown in Fig. 2 ▸ *a*. The 2-sulfate should behave similarly (Fig. 2 ▸ *b*). Jerfy & Roy (1970 ▸) also showed that sulfation of 2-pyridone gives the sulfamate, so that it has not yet been possible to prepare the pyridyl-2-sulfate for comparative purposes. However, a route to 5-hy­droxy­pyridine-2-sulfate together with the isomeric 4- and 6-sulfate esters is known (Behrman & Pitt, 1958 ▸). Examination of these mixed esters by electrophoresis shows that all three are rapidly hydrolyzed under acid catalysis, as predicted by the model. The reaction between potassium per­oxydi­sulfate and 3-hy­droxy­picolinic acid was carried out as usual in KOH solution except that if there is excess per­oxy-di­sulfate, the sulfate ester and the peroxide precipitate from the reaction mixture together: to avoid this the persulfate was used as the limiting reagent. The ester was crystallized from water and 3,6-di­hydroxy­picolinic acid obtained by hydrolysis.

## Structural commentary   

**Figure 3:**
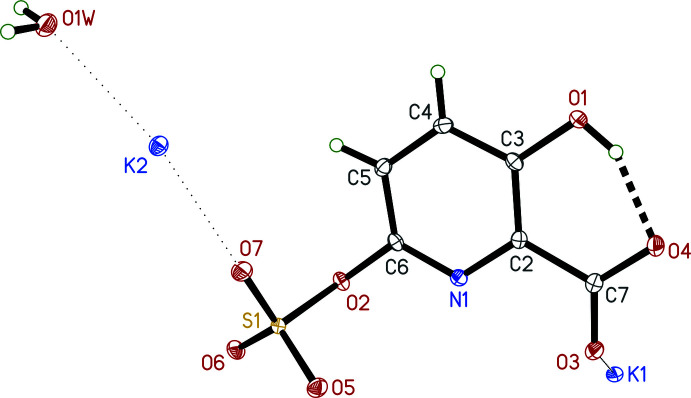
An ellipsoid (50% probability) plot of the asymmetric unit of **I**. The intra­molecular hydrogen bond is shown by the thick dashed line. Dotted lines indicate close contacts of the K^+^ cations.

The asymmetric unit in **I** (Fig. 3 ▸) contains a single pyridine ring with a carboxyl­ate group at the 2-position, a hydroxyl group at the 3-position, and a sulfate group attached to the 6-position. Charge balance is provided by a pair of K^+^ cations. There is also a single water mol­ecule of crystallization present. The carboxyl­ate C—O distances are 1.2510 (16) and 1.2758 (16) Å for C7—O3 and C7—O4, respectively. The longer of these is part of an *S*(6) intra­molecular hydrogen-bonded ring with the hydroxyl group. In the sulfate group, the oxygen atom bound at the 6-position [C6—O2 = 1.3968 (14) Å] is longer [S1—O2 = 1.6343 (9) Å] than the other three S=O bonds [range 1.4361 (9) to 1.4486 (9) Å], as would be expected for this bonding arrangement.

**Figure 4:**
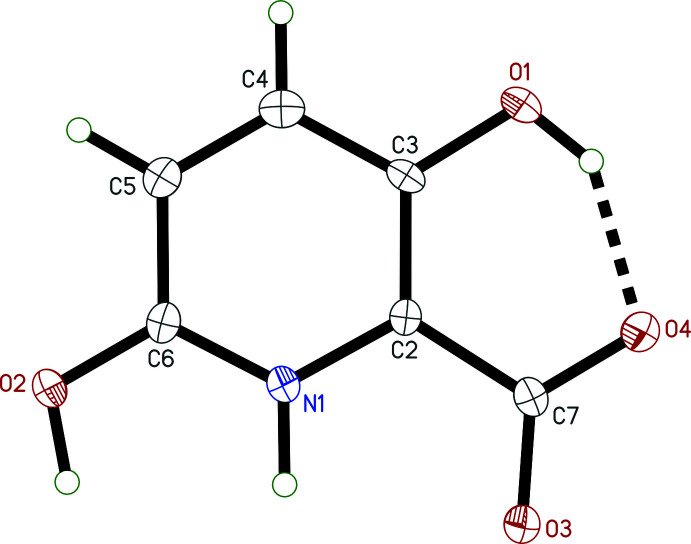
An ellipsoid (50% probability) plot of the asymmetric unit of **II** with the intra­molecular hydrogen bond shown as a thick dashed line.

In **II** (Fig. 4 ▸), the nitro­gen atom of the ring is protonated. Charge balance is provided by the deprotonated 2-carboxyl­ate group [C7—O3 = 1.262 (3), C7—O4 = 1.259 (3) Å], leading to a zwitterionic mol­ecule. The intra­molecular *S*(6) hydrogen-bonded ring from **I** is preserved in **II**. The 6-position of the ring is occupied by a second hydroxyl group. As discussed in more detail in section 6 (*Refinement*), there is a small minor disorder component [refined occupancy 4.7 (3)%] and probable inversion twinning.

## Supra­molecular features   

**Figure 5:**
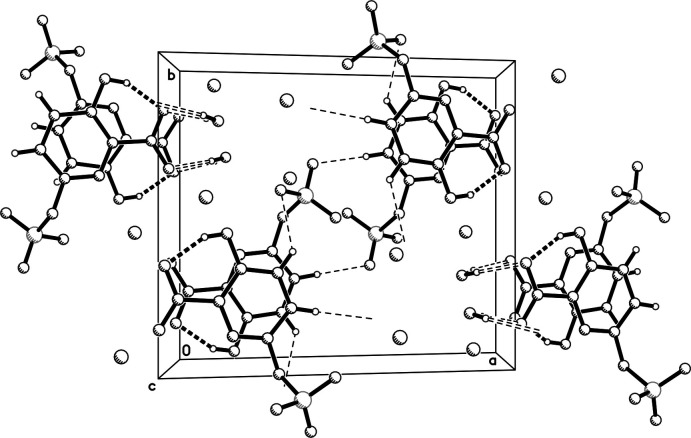
A view of the packing in **I**, viewed down the *c* axis. Intra­molecular hydrogen bonds are shown as thick dashed lines, open dashed lines indicate strong inter­molecular hydrogen bonds, and thin dashed lines denote weaker C—H⋯O inter­actions. Dangling hydrogen bonds extend beyond the depth of the view.

In the packing of **I**, strong O_w_—H⋯O (w = water) hydrogen bonds exist in which the water mol­ecule acts as a linker between *c*-glide-related anions. The water oxygen is also coordinated to the K^+^ cations, both of which are seven coord­inate. Around K1, coordination distances range from 2.7376 (9)–2.9102 (10) Å (for K⋯O) and 2.7869 (11) Å (K1⋯N). For K2, coordination distances range from 2.6769 (9) to 2.9525 (10) Å (all K⋯O). The coordination geometry about each K^+^ cation is very roughly penta­gonal bipyramidal, with K1 much more distorted than K2. As each K^+^ cation is coordinated to the water mol­ecule, the KO_6_N and KO_7_ polyhedra augment the extended chains that propagate parallel to *c* (Fig. 5 ▸, Table 1 ▸). In addition, there are weaker C—H⋯O contacts: pairs of C5—H5⋯O6^ii^ inter­actions form 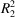
(12) inversion-related dimers and C4—H4⋯O5^i^ contacts link mol­ecules *via c*-glide symmetry (symmetry codes as per Table 1 ▸). This in turn joins *c*-glide-related pyridine rings into an extended π–π stack along the *c*-axis direction. Adjacent rings within the stacks are almost parallel; the dihedral angle being 0.38 (4)° with a centroid–centroid distance of 3.698 (1) Å. These columns of hydrogen-bonded and π-stacked mol­ecules are inter­linked by the aforementioned chains of K^+^ cations and water mol­ecules into a three-dimensional network (Fig. 5 ▸).

**Figure 6:**
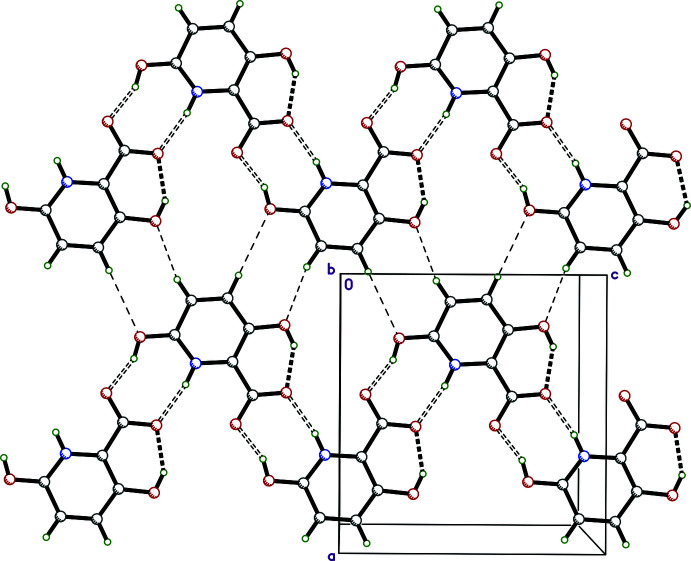
A view of the planar diperiodic network in crystalline **II**, viewed normal to the *ac* plane. Intra­molecular hydrogen bonds are drawn as thick dashed lines, strong inter­molecular hydrogen bonds (O—H⋯O and N—H⋯O) are drawn as double dashed lines. Weaker hydrogen bonds (C—H⋯O) are drawn as thin dashed lines.

The most remarkable feature of the packing in **II** are extended di-periodic hydrogen-bonded networks lying parallel to the *ac* plane (Fig. 6 ▸, Table 2 ▸) that are constrained to *b* = 0.25 (and 0.75) as a consequence of the unusual space-group symmetry of type *Abm*2. Strong O2—H2*O*⋯O3^i^ and N1—H1*N*⋯O4^i^ hydrogen bonds link pairs of 2_1_ screw-related mol­ecules into 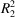
(8) motifs that extend to form ribbons parallel to *c*. Weaker C4—H4⋯O2^iii^ and C5—H5⋯O1^ii^ (symmetry codes as per Table 2 ▸) inter­actions join 2_1_ screw-related ribbons to form the aforementioned networks. Contacts between these planar networks are limited to weak van der Waals inter­actions.

## Database survey   

A search of the Cambridge Structure Database (CSD v5.42, Nov. 2020; Groom *et al.*, 2016 ▸) on a fragment composed of 3-picolinic acid with ‘any non-H’ at the 6-position gave only seven hits. None of these have much in common with **I** or **II**, but the most similar were AGUMEV (ammonium 2,4,6-tri­carb­oxy­pyridine-3-olate monohydrate; Li *et al.*, 2010 ▸) and MAFTEU (6-chloro-3-tri­fluoro­meth­oxy pyridine-2-carb­oxy­lic acid; Manteau *et al.*, 2010 ▸) in that their main components consist of a single pyridine ring with a carb­oxy­lic acid group adjacent to the ring nitro­gen and an oxygen (O^−^ in AGUMEV; O—CF_3_ in MAFTEU) at the 3-position.

Space group *Abm*2 is not common, with only 62 entries listed in v5.42 of the CSD. Excluding polymers, entries flagged with known errors, and those without deposited coordinates left only 47 hits, *i.e*. <0.005% of known structures. Of these, only 29 have *R*_1_ ≤ 5% and none form planar networks in a manner similar to **II**. By any measure, the crystal structure of **II** is unusual.

## Synthesis and crystallization   

*3-Hy­droxy­pyridine-2-carb­oxy­lic acid-6-sulfate dipotassium salt monohydrate (**I**)*: Potassium hydroxide (85%, 1 g, 15 mmol) was dissolved in 10 ml of water and cooled. 3-Hy­droxy­picolinic acid (0.82 g, 6 mmol) followed by potassium per­oxy­disulfate (0.9 g, 3.3 mmol) were added. The reaction mixture was stirred at RT for 24 h. The precipitate was filtered and dried by washing with acetone, yielding 0.46–0.49 g (∼44%) of the dipotassium salt of 3-hy­droxy­pyridine-2-carb­oxy­lic acid-6-sulfate monohydrate (**I**). Compound **I** migrates on paper electrophoresis at pH 7.5 with *R_p_* = 2 as a fluorescent spot. *R_p_* is the migration distance relative to picric acid at *R_p_* = 1 (the starting material has *R_p_* = 1.1). Crystals of **I** grow from aqueous solution when treated as follows: 0.16 g were suspended in 2.0 ml of water, dissolved by heating carefully to about 313 K, and then cooled slowly to RT. Further cooling to 278 K overnight gave 0.12 g of needles. Analysis (%) calculated for C_6_H_5_NO_8_K_2_S: C, 2l.88; H, 1.53; N, 4.25. Found: C, 21.97; H, 1.44; N, 4.25. IR(Nujol): 3485, 3310, 3096, 1701, 1684, 1624, 1574, 1250, 1059, 957, 860, 833, 768, 716, 638 cm^−1^. NMR(D_2_O, 600 MHz) δ 7.27 (*d*, *J* = 8.82 Hz), 7.45 (*d*, *J* = 8.82 Hz). UV (in water): λ_max_, (nm), ɛ (l mol^−1^ cm^−1^): 307, 1120; 230, 1200; 205, 4180. Heating behavior: **I** begins to discolor at about 448 K and then gets darker without melting up to 523 K.

*3,6-Di­hydroxy­picolinic acid (**II**)*: The crude sulfate (**I**, 150 mg), was suspended in 2 ml of water and then heated to about 313 K to dissolve it. HCl was then added to about pH 2. A heavy precipitate formed immediately. After cooling, the colorless solid was filtered and washed with cold water to yield 50–60 mg of the product (yield 67–80%), *R_p_* = 1. The proton NMR spectrum of **II** agreed with that reported by Qiu *et al.* (2019 ▸) except that our spectrum was taken in D_2_O, 600 MHz, δ 6.69 (*d*, *J* = 9.65 Hz) and 7.45 (*d*, *J* = 9.65 Hz). These are shifted because of the solvent difference from Qiu *et al.* (2019 ▸), but the couplings are the same, as is the difference between the two resonances (δ 0.76). 50 mg of **II** was crystallized from 11 ml of hot water under argon to form 35 mg of crystals. Analytical results show that the precipitate is very nearly as clean as the crystals. Analysis (%): calculated for C_6_H_5_NO_4_: C, 46.46; H, 3.25; N, 9.03. Found (crystals), C, 46.22; H, 3.20; N, 9.07. (precipitate), C, 45.65; H, 3.16; N, 9.10. IR: Nujol, 3120, 1614, 1540, 1360, 1269, 1100, 845, 810, 760, 621 cm^−1^. Later, larger crystals were obtained using 88% formic acid as solvent. Upon heating, **II** carbonizes above 473 K without melting. UV (in water): λ_max_, (nm), ɛ (l mol^−1^ cm^−1^) at pH 7: 346, 5970; 243, 4350; 221.5, 9450. Shukla & Kaul (1973 ▸) reported the pH-dependence of the spectrum. [See also Qiu *et al.* (2019 ▸), but on p. S2, line 3, read ‘240’ for ‘360’.]

## Data collection, structure solution and refinement   

The crystals were mounted using polyisobutene oil on the tip of fine glass fibers, which were fastened in copper mounting pins with electrical solder. Crystals of **I** were placed directly into the cold gas stream of a liquid-N_2_ based cryostat, while crystals of **II** were handled using methods developed for macromolecular cryocrystallography (Parkin & Hope, 1998*b* ▸). Diffraction data were collected with the crystals at 90 K. Crystal data, data collection and refinement details are summarized in Table 3 ▸. In **II**, a small minor disorder component was apparent in a difference map. It was modeled by reflection of the major component across a mirror plane perpendicular to its *c* axis, with coordinates related by the mirror plane and constrained by mapping its coordinates to the major component *via SHELXL* FVAR parameters. A test for twinning by inversion using the Flack parameter [*x* = 0.5 (3); Flack & Bernardinelli, 1999 ▸] was indeterminate. The Hooft (Hooft *et al.*, 2008 ▸) and Parsons (Parsons *et al.*, 2013 ▸) parameters [*y* = 0.63 (7); *z* = 0.62 (10), respectively], however, each gave a much stronger suggestion of twinning by inversion. Since the space group itself has a twofold parallel to its *c* axis, this results in a mirror operation (*i.e*. a twofold rotation combined with inversion). Thus, the twinning and disorder in **II** may effectively be treated by the same operation, not unlike that in uric acid dihydrate (Parkin & Hope, 1998*a* ▸; Parkin, 2000 ▸). To ensure satisfactory refinement of disorder, constraints (*SHELXL* command EADP) were used to equalize displacement parameters of superimposed groups. Full occupancy (and major component for **II**) hydrogen atoms were found in difference-Fourier maps. Carbon-bound hydrogen atoms were included using riding models with constrained distances set to 0.95 Å (C*sp* ^2^H). In **I**, the water H atoms were refined but subject to distance and angle–distance restraints, while the hydroxyl H atom was refined freely. In **II**, for O—H and N—H groups, riding models that allowed the bond distance to refine were used. *U*_iso_(H) parameters were set to values of either 1.2*U*_eq_ or 1.5*U*_eq_ (OH only) of the attached atom. The structures were validated using *PLATON* and *checkCIF* (Spek, 2020 ▸).

## Supplementary Material

Crystal structure: contains datablock(s) I, II, global. DOI: 10.1107/S2056989021004904/hb7975sup1.cif

Structure factors: contains datablock(s) I. DOI: 10.1107/S2056989021004904/hb7975Isup2.hkl

Structure factors: contains datablock(s) II. DOI: 10.1107/S2056989021004904/hb7975IIsup3.hkl

Supporting information file. DOI: 10.1107/S2056989021004904/hb7975Isup4.cml

Supporting information file. DOI: 10.1107/S2056989021004904/hb7975IIsup5.cml

CCDC references: 2082685, 2082684

Additional supporting information:  crystallographic information; 3D view; checkCIF report

## Figures and Tables

**Table 1:** Hydrogen-bond geometry (Å, °) for **I** [Chem scheme1]

*D*—H⋯*A*	*D*—H	H⋯*A*	*D*⋯*A*	*D*—H⋯*A*
O1—H1*O*⋯O4	0.843 (19)	1.760 (19)	2.5429 (13)	153.6 (17)
C4—H4⋯O5^i^	0.95	2.64	3.3682 (16)	133
C5—H5⋯O6^ii^	0.95	2.35	3.2943 (15)	175
O1*W*—H1*W*⋯O3^iii^	0.83 (2)	1.97 (2)	2.7852 (13)	165 (2)
O1*W*—H2*W*⋯O4^iv^	0.83 (2)	2.09 (2)	2.8910 (14)	161 (2)

Symmetry codes: (i) x, -y+{\script{1\over 2}}, z-{\script{1\over 2}}; (ii) -x+1, -y+1, -z+1; (iii) x+1, y, z; (iv) x+1, -y+{\script{1\over 2}}, z+{\script{1\over 2}}.

**Table 2:** Hydrogen-bond geometry (Å, °) for **II** [Chem scheme1] The *D*⋯*A* distance for C4—H4⋯O2^iii^ is rather long, but within the bounds noted by Desiraju & Steiner (1999 ▸).

*D*—H⋯*A*	*D*—H	H⋯*A*	*D*⋯*A*	*D*—H⋯*A*
N1—H1*N*⋯O4^i^	0.99	1.77	2.755 (3)	169
O1—H1*O*⋯O4	0.89	1.76	2.535 (3)	145
C5—H5⋯O1^ii^	0.95	2.34	3.269 (3)	166
C4—H4⋯O2^iii^	0.95	2.76	3.712 (4)	180
O2—H2*O*⋯O3^i^	0.94	1.57	2.459 (3)	156
O2—H2*O*⋯O4^i^	0.94	2.56	3.372 (3)	144

Symmetry codes: (i) -x+1, -y+{\script{1\over 2}}, z-{\script{1\over 2}}; (ii) -x, -y+{\script{1\over 2}}, z-{\script{1\over 2}}; (iii) -x, -y+{\script{1\over 2}}, z+{\script{1\over 2}}.

**Table 3:** Experimental details

	**I**	**II**
Crystal data		
Chemical formula	2K^+^·C_6_H_3_NO_7_S^2−^·H_2_O	C_6_H_5_NO_4_
*M*_r_	329.37	155.11
Crystal system, space group	Monoclinic, *P*2_1_/*c*	Orthorhombic, *A* *b* *m*2
Temperature (K)	90	90
*a*, *b*, *c* (Å)	13.3366 (4), 11.5467 (3), 7.3078 (2)	10.2045 (6), 6.1282 (4), 9.7293 (6)
α, β, γ (°)	90, 103.553 (1), 90	90, 90, 90
*V* (Å^3^)	1094.02 (5)	608.42 (7)
*Z*	4	4
Radiation type	Mo *K*α	Cu *K*α
μ (mm^−1^)	1.09	1.27
Crystal size (mm)	0.18 × 0.16 × 0.13	0.14 × 0.10 × 0.02

Data collection		
Diffractometer	Bruker D8 Venture dual source	Bruker D8 Venture dual source
Absorption correction	Multi-scan (*SADABS*; Krause *et al.*, 2015 ▸)	Multi-scan (*SADABS*; Krause *et al.*, 2015 ▸)
*T*_min_, *T*_max_	0.793, 0.862	0.799, 0.971
No. of measured, independent and observed [*I* > 2σ(*I*)] reflections	22347, 2505, 2423	4025, 700, 690
*R*_int_	0.036	0.025
(sin θ/λ)_max_ (Å^−1^)	0.650	0.634

Refinement		
*R*[*F* ^2^ > 2σ(*F* ^2^)], *wR*(*F* ^2^), *S*	0.020, 0.056, 1.08	0.024, 0.068, 1.11
No. of reflections	2505	700
No. of parameters	174	74
No. of restraints	3	4
H-atom treatment	H atoms treated by a mixture of independent and constrained refinement	H atoms treated by a mixture of independent and constrained refinement
Δρ_max_, Δρ_min_ (e Å^−3^)	0.40, −0.44	0.20, −0.25
Absolute structure	–	Refined as a perfect inversion twin
Absolute structure parameter	–	0.5

Computer programs: *APEX3* (Bruker, 2016 ▸), *SHELXT* (Sheldrick, 2015*a* ▸), *SHELXL2018/3* (Sheldrick, 2015*b* ▸), *XP* in *SHELXTL* (Sheldrick, 2008 ▸) and *CIFFIX* (Parkin, 2013 ▸).

## supplementary crystallographic information

### Dipotassium 3-hydroxy-6-(sulfonatooxy)pyridine-2-carboxylate monohydrate (I) Crystal data

**Table:**

2K^+^·C_6_H_3_NO_7_S^2^^−^·H_2_O	*F*(000) = 664
*M**_r_* = 329.37	*D*_x_ = 2.000 Mg m^−^^3^
Monoclinic, *P*2_1_/*c*	Mo *K*α radiation, λ = 0.71073 Å
*a* = 13.3366 (4) Å	Cell parameters from 9459 reflections
*b* = 11.5467 (3) Å	θ = 3.1–27.5°
*c* = 7.3078 (2) Å	µ = 1.09 mm^−^^1^
β = 103.553 (1)°	*T* = 90 K
*V* = 1094.02 (5) Å^3^	Block, colourless
*Z* = 4	0.18 × 0.16 × 0.13 mm

### Dipotassium 3-hydroxy-6-(sulfonatooxy)pyridine-2-carboxylate monohydrate (I) Data collection

**Table:**

Bruker D8 Venture dual source diffractometer	2505 independent reflections
Radiation source: microsource	2423 reflections with *I* > 2σ(*I*)
Detector resolution: 7.41 pixels mm^-1^	*R*_int_ = 0.036
φ and ω scans	θ_max_ = 27.5°, θ_min_ = 1.6°
Absorption correction: multi-scan (*SADABS*; Krause *et al.*, 2015)	*h* = −17→17
*T*_min_ = 0.793, *T*_max_ = 0.862	*k* = −14→14
22347 measured reflections	*l* = −9→9

### Dipotassium 3-hydroxy-6-(sulfonatooxy)pyridine-2-carboxylate monohydrate (I) Refinement

**Table:**

Refinement on *F*^2^	Secondary atom site location: difference Fourier map
Least-squares matrix: full	Hydrogen site location: mixed
*R*[*F*^2^ > 2σ(*F*^2^)] = 0.020	H atoms treated by a mixture of independent and constrained refinement
*wR*(*F*^2^) = 0.056	*w* = 1/[σ^2^(*F*_o_^2^) + (0.0254*P*)^2^ + 0.7213*P*] where *P* = (*F*_o_^2^ + 2*F*_c_^2^)/3
*S* = 1.08	(Δ/σ)_max_ = 0.001
2505 reflections	Δρ_max_ = 0.40 e Å^−^^3^
174 parameters	Δρ_min_ = −0.44 e Å^−^^3^
3 restraints	Extinction correction: SHELXL2018/3 (Sheldrick 2015b), Fc^*^=kFc[1+0.001xFc^2^λ^3^/sin(2θ)]^-1/4^
Primary atom site location: structure-invariant direct methods	Extinction coefficient: 0.0116 (12)

### Dipotassium 3-hydroxy-6-(sulfonatooxy)pyridine-2-carboxylate monohydrate (I) Special details

**Table:**

Experimental. The crystal was mounted using polyisobutene oil on the tip of a fine glass fibre, which was fastened in a copper mounting pin with electrical solder. It was placed directly into the cold gas stream of a liquid-nitrogen based cryostat (Hope, 1994; Parkin & Hope, 1998). Diffraction data were collected with the crystal at 90K, which is standard practice in this laboratory for the majority of flash-cooled crystals.
Geometry. All esds (except the esd in the dihedral angle between two l.s. planes) are estimated using the full covariance matrix. The cell esds are taken into account individually in the estimation of esds in distances, angles and torsion angles; correlations between esds in cell parameters are only used when they are defined by crystal symmetry. An approximate (isotropic) treatment of cell esds is used for estimating esds involving l.s. planes.
Refinement. Refinement progress was checked using *Platon* (Spek, 2020) and by an *R*-tensor (Parkin, 2000). The final model was further checked with the IUCr utility *checkCIF*.

### Dipotassium 3-hydroxy-6-(sulfonatooxy)pyridine-2-carboxylate monohydrate (I) Fractional atomic coordinates and isotropic or equivalent isotropic displacement parameters (Å^2^)

**Table:**

	*x*	*y*	*z*	*U*_iso_*/*U*_eq_	
K1	−0.11303 (2)	0.44196 (2)	0.29817 (4)	0.01068 (9)	
K2	0.65851 (2)	0.37254 (2)	0.80722 (4)	0.01048 (9)	
S1	0.39446 (2)	0.56402 (3)	0.68497 (4)	0.00841 (9)	
O1	0.18689 (8)	0.05598 (7)	0.56350 (13)	0.01168 (19)	
H1O	0.1278 (15)	0.0662 (15)	0.584 (3)	0.018*	
O2	0.32641 (7)	0.49448 (8)	0.50302 (12)	0.00997 (18)	
O3	0.01000 (7)	0.34563 (8)	0.61547 (13)	0.01185 (19)	
O4	0.02411 (7)	0.15242 (8)	0.61413 (13)	0.01227 (19)	
O5	0.32663 (7)	0.58023 (8)	0.81136 (13)	0.01295 (19)	
O6	0.41500 (7)	0.66929 (8)	0.59303 (13)	0.01295 (19)	
O7	0.48251 (7)	0.49241 (8)	0.76027 (14)	0.0149 (2)	
N1	0.20276 (8)	0.37108 (9)	0.55903 (14)	0.0088 (2)	
C2	0.16613 (9)	0.26318 (11)	0.57169 (17)	0.0088 (2)	
C3	0.22393 (10)	0.16468 (11)	0.55284 (17)	0.0093 (2)	
C4	0.32289 (10)	0.17884 (11)	0.52150 (17)	0.0105 (2)	
H4	0.363874	0.113328	0.509029	0.013*	
C5	0.35984 (9)	0.28931 (11)	0.50905 (17)	0.0104 (2)	
H5	0.426568	0.302283	0.487955	0.013*	
C6	0.29543 (10)	0.38126 (10)	0.52862 (17)	0.0089 (2)	
C7	0.05895 (9)	0.25453 (11)	0.60404 (17)	0.0096 (2)	
O1W	0.85262 (7)	0.30218 (9)	0.79832 (14)	0.0151 (2)	
H1W	0.8909 (13)	0.3167 (17)	0.726 (2)	0.023*	
H2W	0.8914 (13)	0.3145 (17)	0.903 (2)	0.023*	

### Dipotassium 3-hydroxy-6-(sulfonatooxy)pyridine-2-carboxylate monohydrate (I) Atomic displacement parameters (Å^2^)

**Table:**

	*U*^11^	*U*^22^	*U*^33^	*U*^12^	*U*^13^	*U*^23^
K1	0.00957 (14)	0.00968 (14)	0.01288 (14)	0.00117 (9)	0.00285 (10)	−0.00086 (9)
K2	0.00967 (14)	0.01126 (14)	0.01074 (14)	0.00044 (9)	0.00281 (10)	0.00146 (9)
S1	0.00738 (15)	0.00825 (15)	0.00966 (15)	−0.00042 (10)	0.00215 (11)	−0.00085 (10)
O1	0.0117 (5)	0.0075 (4)	0.0169 (5)	−0.0005 (3)	0.0054 (4)	0.0000 (3)
O2	0.0121 (4)	0.0089 (4)	0.0087 (4)	−0.0027 (3)	0.0019 (3)	−0.0002 (3)
O3	0.0096 (4)	0.0123 (4)	0.0142 (4)	0.0010 (3)	0.0038 (3)	0.0002 (3)
O4	0.0109 (4)	0.0112 (4)	0.0154 (4)	−0.0017 (3)	0.0044 (3)	0.0009 (3)
O5	0.0123 (4)	0.0164 (5)	0.0111 (4)	−0.0004 (4)	0.0048 (3)	−0.0019 (4)
O6	0.0147 (5)	0.0093 (4)	0.0160 (4)	−0.0028 (3)	0.0059 (4)	−0.0002 (3)
O7	0.0108 (4)	0.0139 (4)	0.0180 (5)	0.0033 (3)	−0.0005 (4)	−0.0017 (4)
N1	0.0096 (5)	0.0093 (5)	0.0072 (5)	−0.0002 (4)	0.0015 (4)	−0.0006 (4)
C2	0.0086 (5)	0.0101 (6)	0.0076 (5)	−0.0008 (4)	0.0018 (4)	0.0005 (4)
C3	0.0112 (6)	0.0082 (6)	0.0078 (5)	−0.0010 (4)	0.0008 (4)	0.0000 (4)
C4	0.0104 (6)	0.0109 (6)	0.0101 (6)	0.0021 (4)	0.0023 (4)	−0.0014 (4)
C5	0.0087 (5)	0.0132 (6)	0.0096 (5)	−0.0002 (5)	0.0026 (4)	−0.0014 (4)
C6	0.0108 (6)	0.0082 (5)	0.0070 (5)	−0.0021 (4)	0.0010 (4)	−0.0001 (4)
C7	0.0091 (6)	0.0129 (6)	0.0065 (5)	−0.0007 (4)	0.0011 (4)	0.0002 (4)
O1W	0.0113 (5)	0.0165 (5)	0.0181 (5)	0.0005 (4)	0.0044 (4)	0.0003 (4)

### Dipotassium 3-hydroxy-6-(sulfonatooxy)pyridine-2-carboxylate monohydrate (I) Geometric parameters (Å, º)

**Table:**

K1—O4^i^	2.7376 (9)	K2—K2^x^	4.6216 (3)
K1—O3	2.7428 (10)	S1—O7	1.4361 (9)
K1—O5^ii^	2.7843 (10)	S1—O6	1.4457 (9)
K1—N1^ii^	2.7869 (11)	S1—O5	1.4486 (9)
K1—O3^ii^	2.8099 (10)	S1—O2	1.6343 (9)
K1—O1W^iii^	2.856 (1)	O1—C3	1.3577 (15)
K1—O1^iv^	2.9102 (10)	O1—H1O	0.843 (19)
K1—S1^ii^	3.7848 (4)	O2—C6	1.3968 (14)
K1—K1^ii^	3.9268 (5)	O3—C7	1.2510 (16)
K1—K2^v^	4.2041 (4)	O4—C7	1.2758 (16)
K1—K2^iii^	4.7508 (4)	N1—C6	1.3112 (16)
K2—O7	2.6769 (9)	N1—C2	1.3492 (16)
K2—O6^vi^	2.7084 (10)	C2—C3	1.3988 (17)
K2—O1W	2.7286 (10)	C2—C7	1.5057 (17)
K2—O2^vii^	2.7842 (9)	C3—C4	1.4010 (17)
K2—O5^viii^	2.8012 (9)	C4—C5	1.3781 (18)
K2—O6^vii^	2.8993 (10)	C4—H4	0.9500
K2—O1^ix^	2.9525 (10)	C5—C6	1.3940 (17)
K2—S1^vii^	3.5751 (4)	C5—H5	0.9500
K2—S1^vi^	3.6348 (4)	O1W—H1W	0.832 (15)
K2—K2^i^	4.6216 (3)	O1W—H2W	0.831 (15)
			
O4^i^—K1—O3	85.28 (3)	O5^viii^—K2—K1^xi^	41.02 (2)
O4^i^—K1—O5^ii^	125.34 (3)	O6^vii^—K2—K1^xi^	154.00 (2)
O3—K1—O5^ii^	125.08 (3)	O1^ix^—K2—K1^xi^	43.784 (19)
O4^i^—K1—N1^ii^	152.53 (3)	S1^vii^—K2—K1^xi^	138.793 (10)
O3—K1—N1^ii^	102.82 (3)	S1^vi^—K2—K1^xi^	106.147 (8)
O5^ii^—K1—N1^ii^	71.26 (3)	O7—K2—K2^i^	112.19 (2)
O4^i^—K1—O3^ii^	96.31 (3)	O6^vi^—K2—K2^i^	75.48 (2)
O3—K1—O3^ii^	90.00 (3)	O1W—K2—K2^i^	67.86 (2)
O5^ii^—K1—O3^ii^	123.94 (3)	O2^vii^—K2—K2^i^	71.370 (18)
N1^ii^—K1—O3^ii^	58.00 (3)	O5^viii^—K2—K2^i^	153.22 (2)
O4^i^—K1—O1W^iii^	74.51 (3)	O6^vii^—K2—K2^i^	33.178 (19)
O3—K1—O1W^iii^	70.34 (3)	O1^ix^—K2—K2^i^	124.254 (19)
O5^ii^—K1—O1W^iii^	75.87 (3)	S1^vii^—K2—K2^i^	50.703 (5)
N1^ii^—K1—O1W^iii^	132.96 (3)	S1^vi^—K2—K2^i^	56.517 (8)
O3^ii^—K1—O1W^iii^	158.67 (3)	K1^xi^—K2—K2^i^	131.084 (6)
O4^i^—K1—O1^iv^	81.77 (3)	O7—K2—K2^x^	104.80 (2)
O3—K1—O1^iv^	163.61 (3)	O6^vi^—K2—K2^x^	35.86 (2)
O5^ii^—K1—O1^iv^	71.02 (3)	O1W—K2—K2^x^	90.71 (2)
N1^ii^—K1—O1^iv^	84.60 (3)	O2^vii^—K2—K2^x^	174.19 (2)
O3^ii^—K1—O1^iv^	81.53 (3)	O5^viii^—K2—K2^x^	49.13 (2)
O1W^iii^—K1—O1^iv^	115.26 (3)	O6^vii^—K2—K2^x^	129.27 (2)
O4^i^—K1—S1^ii^	142.24 (2)	O1^ix^—K2—K2^x^	108.403 (19)
O3—K1—S1^ii^	111.50 (2)	S1^vii^—K2—K2^x^	152.157 (10)
O5^ii^—K1—S1^ii^	18.571 (19)	S1^vi^—K2—K2^x^	49.568 (8)
N1^ii^—K1—S1^ii^	59.06 (2)	K1^xi^—K2—K2^x^	64.932 (5)
O3^ii^—K1—S1^ii^	116.43 (2)	K2^i^—K2—K2^x^	104.485 (11)
O1W^iii^—K1—S1^ii^	79.72 (2)	O7—S1—O6	115.80 (6)
O1^iv^—K1—S1^ii^	84.87 (2)	O7—S1—O5	114.01 (6)
O4^i^—K1—K1^ii^	91.22 (2)	O6—S1—O5	113.72 (6)
O3—K1—K1^ii^	45.69 (2)	O7—S1—O2	106.09 (5)
O5^ii^—K1—K1^ii^	143.23 (2)	O6—S1—O2	99.47 (5)
N1^ii^—K1—K1^ii^	77.04 (2)	O5—S1—O2	105.75 (5)
O3^ii^—K1—K1^ii^	44.305 (19)	O7—S1—K2^vii^	117.03 (4)
O1W^iii^—K1—K1^ii^	115.57 (2)	O6—S1—K2^vii^	51.28 (4)
O1^iv^—K1—K1^ii^	124.38 (2)	O5—S1—K2^vii^	127.54 (4)
S1^ii^—K1—K1^ii^	125.061 (11)	O2—S1—K2^vii^	48.78 (3)
O4^i^—K1—K2^v^	86.50 (2)	O7—S1—K2^ix^	134.65 (4)
O3—K1—K2^v^	144.54 (2)	O6—S1—K2^ix^	40.71 (4)
O5^ii^—K1—K2^v^	41.328 (19)	O5—S1—K2^ix^	73.18 (4)
N1^ii^—K1—K2^v^	100.27 (2)	O2—S1—K2^ix^	114.92 (3)
O3^ii^—K1—K2^v^	125.21 (2)	K2^vii^—S1—K2^ix^	79.729 (8)
O1W^iii^—K1—K2^v^	74.21 (2)	O7—S1—K1^ii^	134.83 (4)
O1^iv^—K1—K2^v^	44.587 (19)	O6—S1—K1^ii^	109.13 (4)
S1^ii^—K1—K2^v^	59.892 (7)	O5—S1—K1^ii^	37.75 (4)
K1^ii^—K1—K2^v^	168.952 (12)	O2—S1—K1^ii^	69.61 (3)
O4^i^—K1—K2^iii^	101.95 (2)	K2^vii^—S1—K1^ii^	94.176 (9)
O3—K1—K2^iii^	86.38 (2)	K2^ix^—S1—K1^ii^	79.600 (8)
O5^ii^—K1—K2^iii^	46.95 (2)	C3—O1—K1^xii^	114.60 (7)
N1^ii^—K1—K2^iii^	104.70 (2)	C3—O1—K2^vi^	116.45 (7)
O3^ii^—K1—K2^iii^	161.00 (2)	K1^xii^—O1—K2^vi^	91.63 (3)
O1W^iii^—K1—K2^iii^	30.91 (2)	C3—O1—H1O	104.4 (12)
O1^iv^—K1—K2^iii^	106.07 (2)	K1^xii^—O1—H1O	95.2 (12)
S1^ii^—K1—K2^iii^	48.809 (6)	K2^vi^—O1—H1O	131.0 (12)
K1^ii^—K1—K2^iii^	129.234 (11)	C6—O2—S1	118.44 (7)
K2^v^—K1—K2^iii^	61.786 (6)	C6—O2—K2^vii^	134.86 (7)
O7—K2—O6^vi^	96.93 (3)	S1—O2—K2^vii^	105.02 (4)
O7—K2—O1W	163.59 (3)	C7—O3—K1	120.18 (8)
O6^vi^—K2—O1W	98.84 (3)	C7—O3—K1^ii^	120.90 (8)
O7—K2—O2^vii^	80.73 (3)	K1—O3—K1^ii^	90.00 (3)
O6^vi^—K2—O2^vii^	142.83 (3)	C7—O4—K1^x^	133.68 (8)
O1W—K2—O2^vii^	83.96 (3)	S1—O5—K1^ii^	123.68 (5)
O7—K2—O5^viii^	83.24 (3)	S1—O5—K2^viii^	138.63 (5)
O6^vi^—K2—O5^viii^	81.17 (3)	K1^ii^—O5—K2^viii^	97.65 (3)
O1W—K2—O5^viii^	103.61 (3)	S1—O6—K2^ix^	118.92 (5)
O2^vii^—K2—O5^viii^	134.52 (3)	S1—O6—K2^vii^	105.82 (5)
O7—K2—O6^vii^	82.98 (3)	K2^ix^—O6—K2^vii^	110.96 (3)
O6^vi^—K2—O6^vii^	93.96 (2)	S1—O7—K2	163.84 (6)
O1W—K2—O6^vii^	91.42 (3)	C6—N1—C2	117.72 (11)
O2^vii^—K2—O6^vii^	48.87 (3)	C6—N1—K1^ii^	119.90 (8)
O5^viii^—K2—O6^vii^	164.72 (3)	C2—N1—K1^ii^	119.70 (8)
O7—K2—O1^ix^	101.29 (3)	N1—C2—C3	121.82 (11)
O6^vi^—K2—O1^ix^	143.63 (3)	N1—C2—C7	116.38 (11)
O1W—K2—O1^ix^	68.03 (3)	C3—C2—C7	121.79 (11)
O2^vii^—K2—O1^ix^	71.74 (3)	O1—C3—C2	121.99 (11)
O5^viii^—K2—O1^ix^	70.16 (3)	O1—C3—C4	119.11 (11)
O6^vii^—K2—O1^ix^	119.21 (3)	C2—C3—C4	118.90 (11)
O7—K2—S1^vii^	78.73 (2)	C5—C4—C3	118.94 (11)
O6^vi^—K2—S1^vii^	116.72 (2)	C5—C4—H4	120.5
O1W—K2—S1^vii^	90.11 (2)	C3—C4—H4	120.5
O2^vii^—K2—S1^vii^	26.201 (18)	C4—C5—C6	117.37 (11)
O5^viii^—K2—S1^vii^	155.84 (2)	C4—C5—H5	121.3
O6^vii^—K2—S1^vii^	22.895 (18)	C6—C5—H5	121.3
O1^ix^—K2—S1^vii^	97.67 (2)	N1—C6—C5	125.24 (11)
O7—K2—S1^vi^	109.94 (2)	N1—C6—O2	115.26 (11)
O6^vi^—K2—S1^vi^	20.38 (2)	C5—C6—O2	119.36 (11)
O1W—K2—S1^vi^	84.12 (2)	O3—C7—O4	124.78 (11)
O2^vii^—K2—S1^vi^	127.21 (2)	O3—C7—C2	118.96 (11)
O5^viii^—K2—S1^vi^	98.27 (2)	O4—C7—C2	116.25 (11)
O6^vii^—K2—S1^vi^	80.302 (19)	K2—O1W—K1^xiii^	116.56 (4)
O1^ix^—K2—S1^vi^	145.27 (2)	K2—O1W—H1W	132.9 (13)
S1^vii^—K2—S1^vi^	102.902 (10)	K1^xiii^—O1W—H1W	94.4 (14)
O7—K2—K1^xi^	116.69 (2)	K2—O1W—H2W	108.4 (13)
O6^vi^—K2—K1^xi^	99.86 (2)	K1^xiii^—O1W—H2W	96.0 (14)
O1W—K2—K1^xi^	64.86 (2)	H1W—O1W—H2W	102.0 (15)
O2^vii^—K2—K1^xi^	114.43 (2)		
			
O7—S1—O2—C6	55.28 (10)	K1^ii^—S1—O7—K2	142.06 (18)
O6—S1—O2—C6	175.77 (9)	C6—N1—C2—C3	−0.18 (17)
O5—S1—O2—C6	−66.14 (10)	K1^ii^—N1—C2—C3	−161.62 (9)
K2^vii^—S1—O2—C6	167.38 (11)	C6—N1—C2—C7	−179.2 (1)
K2^ix^—S1—O2—C6	−144.57 (8)	K1^ii^—N1—C2—C7	19.36 (14)
K1^ii^—S1—O2—C6	−77.22 (8)	K1^xii^—O1—C3—C2	101.95 (11)
O7—S1—O2—K2^vii^	−112.10 (5)	K2^vi^—O1—C3—C2	−152.82 (9)
O6—S1—O2—K2^vii^	8.39 (5)	K1^xii^—O1—C3—C4	−77.87 (12)
O5—S1—O2—K2^vii^	126.48 (5)	K2^vi^—O1—C3—C4	27.36 (14)
K2^ix^—S1—O2—K2^vii^	48.05 (4)	N1—C2—C3—O1	−179.28 (11)
K1^ii^—S1—O2—K2^vii^	115.40 (3)	C7—C2—C3—O1	−0.31 (18)
O7—S1—O5—K1^ii^	−133.28 (6)	N1—C2—C3—C4	0.54 (18)
O6—S1—O5—K1^ii^	90.99 (7)	C7—C2—C3—C4	179.51 (11)
O2—S1—O5—K1^ii^	−17.12 (7)	O1—C3—C4—C5	179.41 (11)
K2^vii^—S1—O5—K1^ii^	32.58 (8)	C2—C3—C4—C5	−0.41 (18)
K2^ix^—S1—O5—K1^ii^	94.73 (5)	C3—C4—C5—C6	−0.03 (18)
O7—S1—O5—K2^viii^	43.85 (10)	C2—N1—C6—C5	−0.32 (18)
O6—S1—O5—K2^viii^	−91.88 (9)	K1^ii^—N1—C6—C5	161.09 (9)
O2—S1—O5—K2^viii^	160.01 (7)	C2—N1—C6—O2	175.49 (10)
K2^vii^—S1—O5—K2^viii^	−150.29 (5)	K1^ii^—N1—C6—O2	−23.10 (13)
K2^ix^—S1—O5—K2^viii^	−88.14 (7)	C4—C5—C6—N1	0.42 (19)
K1^ii^—S1—O5—K2^viii^	177.13 (12)	C4—C5—C6—O2	−175.23 (11)
O7—S1—O6—K2^ix^	−129.41 (6)	S1—O2—C6—N1	94.96 (11)
O5—S1—O6—K2^ix^	5.50 (8)	K2^vii^—O2—C6—N1	−102.36 (12)
O2—S1—O6—K2^ix^	117.46 (5)	S1—O2—C6—C5	−88.96 (12)
K2^vii^—S1—O6—K2^ix^	125.55 (7)	K2^vii^—O2—C6—C5	73.72 (14)
K1^ii^—S1—O6—K2^ix^	45.88 (6)	K1—O3—C7—O4	−88.64 (13)
O7—S1—O6—K2^vii^	105.04 (6)	K1^ii^—O3—C7—O4	160.99 (9)
O5—S1—O6—K2^vii^	−120.05 (5)	K1—O3—C7—C2	89.91 (11)
O2—S1—O6—K2^vii^	−8.09 (5)	K1^ii^—O3—C7—C2	−20.46 (14)
K2^ix^—S1—O6—K2^vii^	−125.55 (7)	K1^x^—O4—C7—O3	−30.63 (18)
K1^ii^—S1—O6—K2^vii^	−79.67 (4)	K1^x^—O4—C7—C2	150.78 (8)
O6—S1—O7—K2	−44.2 (2)	N1—C2—C7—O3	0.67 (17)
O5—S1—O7—K2	−179.00 (19)	C3—C2—C7—O3	−178.36 (11)
O2—S1—O7—K2	65.0 (2)	N1—C2—C7—O4	179.34 (10)
K2^vii^—S1—O7—K2	13.6 (2)	C3—C2—C7—O4	0.31 (17)
K2^ix^—S1—O7—K2	−89.3 (2)		

Symmetry codes: (i) *x*, −*y*+1/2, *z*−1/2; (ii) −*x*, −*y*+1, −*z*+1; (iii) *x*−1, −*y*+1/2, *z*−1/2; (iv) −*x*, *y*+1/2, −*z*+1/2; (v) *x*−1, *y*, *z*−1; (vi) −*x*+1, *y*−1/2, −*z*+3/2; (vii) −*x*+1, −*y*+1, −*z*+1; (viii) −*x*+1, −*y*+1, −*z*+2; (ix) −*x*+1, *y*+1/2, −*z*+3/2; (x) *x*, −*y*+1/2, *z*+1/2; (xi) *x*+1, *y*, *z*+1; (xii) −*x*, *y*−1/2, −*z*+1/2; (xiii) *x*+1, −*y*+1/2, *z*+1/2.

### Dipotassium 3-hydroxy-6-(sulfonatooxy)pyridine-2-carboxylate monohydrate (I) Hydrogen-bond geometry (Å, º)

**Table:**

*D*—H···*A*	*D*—H	H···*A*	*D*···*A*	*D*—H···*A*
O1—H1*O*···O4	0.843 (19)	1.760 (19)	2.5429 (13)	153.6 (17)
C4—H4···O5^i^	0.95	2.64	3.3682 (16)	133
C5—H5···O6^vii^	0.95	2.35	3.2943 (15)	175
O1*W*—H1*W*···O3^xiv^	0.83 (2)	1.97 (2)	2.7852 (13)	165 (2)
O1*W*—H2*W*···O4^xiii^	0.83 (2)	2.09 (2)	2.8910 (14)	161 (2)

Symmetry codes: (i) *x*, −*y*+1/2, *z*−1/2; (vii) −*x*+1, −*y*+1, −*z*+1; (xiii) *x*+1, −*y*+1/2, *z*+1/2; (xiv) *x*+1, *y*, *z*.

### 3-Hydroxy-6-oxo-1,6-dihydropyridine-2-carboxylic acid (II) Crystal data

**Table:**

C_6_H_5_NO_4_	*D*_x_ = 1.693 Mg m^−^^3^
*M**_r_* = 155.11	Cu *K*α radiation, λ = 1.54178 Å
Orthorhombic, *A**b**m*2	Cell parameters from 3820 reflections
*a* = 10.2045 (6) Å	θ = 4.3–78.5°
*b* = 6.1282 (4) Å	µ = 1.27 mm^−^^1^
*c* = 9.7293 (6) Å	*T* = 90 K
*V* = 608.42 (7) Å^3^	Plate, colourless
*Z* = 4	0.14 × 0.10 × 0.02 mm
*F*(000) = 320	

### 3-Hydroxy-6-oxo-1,6-dihydropyridine-2-carboxylic acid (II) Data collection

**Table:**

Bruker D8 Venture dual source diffractometer	700 independent reflections
Radiation source: microsource	690 reflections with *I* > 2σ(*I*)
Detector resolution: 7.41 pixels mm^-1^	*R*_int_ = 0.025
φ and ω scans	θ_max_ = 77.8°, θ_min_ = 4.3°
Absorption correction: multi-scan (*SADABS*; Krause *et al.*, 2015)	*h* = −11→12
*T*_min_ = 0.799, *T*_max_ = 0.971	*k* = −7→7
4025 measured reflections	*l* = −11→12

### 3-Hydroxy-6-oxo-1,6-dihydropyridine-2-carboxylic acid (II) Refinement

**Table:**

Refinement on *F*^2^	Secondary atom site location: difference Fourier map
Least-squares matrix: full	Hydrogen site location: difference Fourier map
*R*[*F*^2^ > 2σ(*F*^2^)] = 0.024	H atoms treated by a mixture of independent and constrained refinement
*wR*(*F*^2^) = 0.068	*w* = 1/[σ^2^(*F*_o_^2^) + (0.050*P*)^2^ + 0.0512*P*] where *P* = (*F*_o_^2^ + 2*F*_c_^2^)/3
*S* = 1.11	(Δ/σ)_max_ = 0.024
700 reflections	Δρ_max_ = 0.20 e Å^−^^3^
74 parameters	Δρ_min_ = −0.25 e Å^−^^3^
4 restraints	Absolute structure: Refined as a perfect inversion twin
Primary atom site location: structure-invariant direct methods	Absolute structure parameter: 0.5

### 3-Hydroxy-6-oxo-1,6-dihydropyridine-2-carboxylic acid (II) Special details

**Table:**

Experimental. The crystal was mounted using polyisobutene oil on the tip of a fine glass fibre, which was fastened in a copper mounting pin with electrical solder. It was placed directly into the cold gas stream of a liquid-nitrogen based cryostat (Hope, 1994; Parkin & Hope, 1998). Diffraction data were collected with the crystal at 90K, which is standard practice in this laboratory for the majority of flash-cooled crystals.
Geometry. All esds (except the esd in the dihedral angle between two l.s. planes) are estimated using the full covariance matrix. The cell esds are taken into account individually in the estimation of esds in distances, angles and torsion angles; correlations between esds in cell parameters are only used when they are defined by crystal symmetry. An approximate (isotropic) treatment of cell esds is used for estimating esds involving l.s. planes.
Refinement. Refined as a two-component perfect inversion twin.

### 3-Hydroxy-6-oxo-1,6-dihydropyridine-2-carboxylic acid (II) Fractional atomic coordinates and isotropic or equivalent isotropic displacement parameters (Å^2^)

**Table:**

	*x*	*y*	*z*	*U*_iso_*/*U*_eq_	Occ. (<1)
N1	0.3327 (2)	0.250000	0.44103 (19)	0.0146 (4)	0.953 (3)
H1N	0.421 (3)	0.25000 (2)	0.3976 (15)	0.017*	0.953 (3)
C2	0.3260 (2)	0.250000	0.5827 (2)	0.0131 (5)	0.953 (3)
C3	0.2033 (3)	0.250000	0.6458 (3)	0.0148 (5)	0.953 (3)
O1	0.19022 (19)	0.250000	0.7831 (2)	0.0188 (4)	0.953 (3)
H1O	0.269 (4)	0.25000 (2)	0.8219 (19)	0.028*	0.953 (3)
C4	0.0919 (3)	0.250000	0.5626 (3)	0.0194 (6)	0.953 (3)
H4	0.007769	0.250000	0.604343	0.023*	0.953 (3)
C5	0.1011 (2)	0.250000	0.4226 (3)	0.0191 (5)	0.953 (3)
H5	0.024166	0.250000	0.367711	0.023*	0.953 (3)
C6	0.2280 (2)	0.250000	0.3592 (3)	0.0154 (5)	0.953 (3)
O2	0.2369 (2)	0.250000	0.22580 (18)	0.0186 (4)	0.953 (3)
H2O	0.326 (3)	0.25000 (2)	0.200 (1)	0.028*	0.953 (3)
C7	0.4488 (2)	0.250000	0.6616 (2)	0.0158 (5)	0.953 (3)
O3	0.55590 (18)	0.250000	0.59684 (19)	0.0182 (4)	0.953 (3)
O4	0.43850 (17)	0.250000	0.7906 (2)	0.0224 (5)	0.953 (3)
N1'	0.3327 (2)	0.250000	0.55897 (19)	0.0131 (5)	0.047 (3)
H1'N	0.421 (3)	0.25000 (2)	0.6024 (16)	0.016*	0.047 (3)
C2'	0.3260 (2)	0.250000	0.4173 (2)	0.0146 (4)	0.047 (3)
C3'	0.2033 (3)	0.250000	0.3542 (3)	0.0154 (5)	0.047 (3)
O1'	0.19022 (19)	0.250000	0.2169 (2)	0.0186 (4)	0.047 (3)
H1'O	0.269 (4)	0.25000 (2)	0.178 (2)	0.028*	0.047 (3)
C4'	0.0919 (3)	0.250000	0.4374 (3)	0.0191 (5)	0.047 (3)
H4'	0.007769	0.250000	0.395657	0.023*	0.047 (3)
C5'	0.1011 (2)	0.250000	0.5774 (3)	0.0194 (6)	0.047 (3)
H5'	0.024166	0.250000	0.632289	0.023*	0.047 (3)
C6'	0.2280 (2)	0.250000	0.6408 (3)	0.0148 (5)	0.047 (3)
O2'	0.2369 (2)	0.250000	0.77420 (18)	0.0188 (4)	0.047 (3)
H2'O	0.326 (4)	0.25000 (2)	0.8001 (11)	0.028*	0.047 (3)
C7'	0.4488 (2)	0.250000	0.3384 (2)	0.0158 (5)	0.047 (3)
O3'	0.55590 (18)	0.250000	0.40316 (19)	0.0182 (4)	0.047 (3)
O4'	0.43850 (17)	0.250000	0.2094 (2)	0.0224 (5)	0.047 (3)

### 3-Hydroxy-6-oxo-1,6-dihydropyridine-2-carboxylic acid (II) Atomic displacement parameters (Å^2^)

**Table:**

	*U*^11^	*U*^22^	*U*^33^	*U*^12^	*U*^13^	*U*^23^
N1	0.0142 (9)	0.0190 (8)	0.0105 (11)	0.000	0.0000 (9)	0.000
C2	0.0127 (12)	0.0155 (9)	0.0112 (10)	0.000	−0.0016 (9)	0.000
C3	0.0151 (10)	0.0180 (11)	0.0113 (11)	0.000	0.0040 (8)	0.000
O1	0.0167 (9)	0.0274 (9)	0.0123 (8)	0.000	0.0041 (7)	0.000
C4	0.0159 (12)	0.0222 (10)	0.0202 (13)	0.000	0.0025 (10)	0.000
C5	0.0150 (11)	0.0259 (10)	0.0166 (10)	0.000	−0.0017 (9)	0.000
C6	0.0150 (12)	0.017 (1)	0.0141 (12)	0.000	−0.0031 (9)	0.000
O2	0.0163 (9)	0.0295 (8)	0.0101 (8)	0.000	0.0003 (6)	0.000
C7	0.0167 (10)	0.0196 (12)	0.0112 (14)	0.000	−0.0002 (9)	0.000
O3	0.0141 (8)	0.0268 (7)	0.0137 (8)	0.000	−0.0008 (6)	0.000
O4	0.0148 (12)	0.0368 (11)	0.0157 (9)	0.000	−0.0014 (7)	0.000
N1'	0.0127 (12)	0.0155 (9)	0.0112 (10)	0.000	−0.0016 (9)	0.000
C2'	0.0142 (9)	0.0190 (8)	0.0105 (11)	0.000	0.0000 (9)	0.000
C3'	0.0150 (12)	0.017 (1)	0.0141 (12)	0.000	−0.0031 (9)	0.000
O1'	0.0163 (9)	0.0295 (8)	0.0101 (8)	0.000	0.0003 (6)	0.000
C4'	0.0150 (11)	0.0259 (10)	0.0166 (10)	0.000	−0.0017 (9)	0.000
C5'	0.0159 (12)	0.0222 (10)	0.0202 (13)	0.000	0.0025 (10)	0.000
C6'	0.0151 (10)	0.0180 (11)	0.0113 (11)	0.000	0.0040 (8)	0.000
O2'	0.0167 (9)	0.0274 (9)	0.0123 (8)	0.000	0.0041 (7)	0.000
C7'	0.0167 (10)	0.0196 (12)	0.0112 (14)	0.000	−0.0002 (9)	0.000
O3'	0.0141 (8)	0.0268 (7)	0.0137 (8)	0.000	−0.0008 (6)	0.000
O4'	0.0148 (12)	0.0368 (11)	0.0157 (9)	0.000	−0.0014 (7)	0.000

### 3-Hydroxy-6-oxo-1,6-dihydropyridine-2-carboxylic acid (II) Geometric parameters (Å, º)

**Table:**

N1—C6	1.333 (3)	N1'—C6'	1.333 (3)
N1—C2	1.381 (3)	N1'—C2'	1.381 (3)
N1—H1N	0.99 (3)	N1'—H1'N	0.99 (4)
C2—C3	1.394 (3)	C2'—C3'	1.394 (3)
C2—C7	1.470 (3)	C2'—C7'	1.470 (3)
C3—O1	1.342 (3)	C3'—O1'	1.342 (3)
C3—C4	1.395 (4)	C3'—C4'	1.395 (4)
O1—H1O	0.89 (4)	O1'—H1'O	0.89 (4)
C4—C5	1.365 (3)	C4'—C5'	1.365 (3)
C4—H4	0.9500	C4'—H4'	0.9500
C5—C6	1.434 (3)	C5'—C6'	1.434 (3)
C5—H5	0.9500	C5'—H5'	0.9500
C6—O2	1.301 (3)	C6'—O2'	1.301 (3)
O2—H2O	0.94 (4)	O2'—H2'O	0.94 (4)
C7—O4	1.259 (3)	C7'—O4'	1.259 (3)
C7—O3	1.262 (3)	C7'—O3'	1.262 (3)
			
C6—N1—C2	123.81 (19)	C6'—N1'—C2'	123.81 (19)
C6—N1—H1N	118.1	C6'—N1'—H1'N	118.1
C2—N1—H1N	118.1	C2'—N1'—H1'N	118.1
N1—C2—C3	119.0 (2)	N1'—C2'—C3'	119.0 (2)
N1—C2—C7	118.6 (2)	N1'—C2'—C7'	118.6 (2)
C3—C2—C7	122.41 (19)	C3'—C2'—C7'	122.41 (19)
O1—C3—C2	121.8 (2)	O1'—C3'—C2'	121.8 (2)
O1—C3—C4	119.8 (2)	O1'—C3'—C4'	119.8 (2)
C2—C3—C4	118.4 (2)	C2'—C3'—C4'	118.4 (2)
C3—O1—H1O	109.5	C3'—O1'—H1'O	109.5
C5—C4—C3	121.5 (2)	C5'—C4'—C3'	121.5 (2)
C5—C4—H4	119.2	C5'—C4'—H4'	119.2
C3—C4—H4	119.2	C3'—C4'—H4'	119.2
C4—C5—C6	119.5 (2)	C4'—C5'—C6'	119.5 (2)
C4—C5—H5	120.3	C4'—C5'—H5'	120.3
C6—C5—H5	120.3	C6'—C5'—H5'	120.3
O2—C6—N1	122.7 (2)	O2'—C6'—N1'	122.7 (2)
O2—C6—C5	119.5 (2)	O2'—C6'—C5'	119.5 (2)
N1—C6—C5	117.8 (2)	N1'—C6'—C5'	117.8 (2)
C6—O2—H2O	109.5	C6'—O2'—H2'O	109.5
O4—C7—O3	124.8 (2)	O4'—C7'—O3'	124.8 (2)
O4—C7—C2	116.7 (2)	O4'—C7'—C2'	116.7 (2)
O3—C7—C2	118.53 (19)	O3'—C7'—C2'	118.53 (19)
			
C6—N1—C2—C3	0.000 (1)	C6'—N1'—C2'—C3'	0.000 (1)
C6—N1—C2—C7	180.000 (1)	C6'—N1'—C2'—C7'	180.000 (1)
N1—C2—C3—O1	180.000 (1)	N1'—C2'—C3'—O1'	180.000 (1)
C7—C2—C3—O1	0.000 (1)	C7'—C2'—C3'—O1'	0.000 (1)
N1—C2—C3—C4	0.000 (1)	N1'—C2'—C3'—C4'	0.000 (1)
C7—C2—C3—C4	180.000 (1)	C7'—C2'—C3'—C4'	180.000 (1)
O1—C3—C4—C5	180.000 (1)	O1'—C3'—C4'—C5'	180.000 (1)
C2—C3—C4—C5	0.000 (1)	C2'—C3'—C4'—C5'	0.000 (1)
C3—C4—C5—C6	0.000 (1)	C3'—C4'—C5'—C6'	0.000 (1)
C2—N1—C6—O2	180.000 (1)	C2'—N1'—C6'—O2'	180.000 (1)
C2—N1—C6—C5	0.000 (1)	C2'—N1'—C6'—C5'	0.000 (1)
C4—C5—C6—O2	180.000 (1)	C4'—C5'—C6'—O2'	180.000 (1)
C4—C5—C6—N1	0.000 (1)	C4'—C5'—C6'—N1'	0.000 (1)
N1—C2—C7—O4	180.000 (1)	N1'—C2'—C7'—O4'	180.000 (1)
C3—C2—C7—O4	0.000 (1)	C3'—C2'—C7'—O4'	0.000 (1)
N1—C2—C7—O3	0.000 (1)	N1'—C2'—C7'—O3'	0.000 (1)
C3—C2—C7—O3	180.000 (1)	C3'—C2'—C7'—O3'	180.000 (1)

### 3-Hydroxy-6-oxo-1,6-dihydropyridine-2-carboxylic acid (II) Hydrogen-bond geometry (Å, º)

The *D*···*A* distance for C4—H4···O2^iii^ is rather long, but within the bounds noted by Desiraju & Steiner (1999).

**Table:**

*D*—H···*A*	*D*—H	H···*A*	*D*···*A*	*D*—H···*A*
N1—H1*N*···O4^i^	0.99	1.77	2.755 (3)	169
O1—H1*O*···O4	0.89	1.76	2.535 (3)	145
C5—H5···O1^ii^	0.95	2.34	3.269 (3)	166
C4—H4···O2^iii^	0.95	2.76	3.712 (4)	180
O2—H2*O*···O3^i^	0.94	1.57	2.459 (3)	156
O2—H2*O*···O4^i^	0.94	2.56	3.372 (3)	144

Symmetry codes: (i) −*x*+1, −*y*+1/2, *z*−1/2; (ii) −*x*, −*y*+1/2, *z*−1/2; (iii) −*x*, −*y*+1/2, *z*+1/2.

## References

[bb1] Behrman, E. J. (1988). *Org. React.* **35**, 421–511.

[bb2] Behrman, E. J. (2021). *Mini-Rev. Org. Chem.* **18**, 621–625.

[bb3] Behrman, E. J. & Pitt, B. M. (1958). *J. Am. Chem. Soc.* **80**, 3717–3718.

[bb4] Benkovic, S. J. (1966). *J. Am. Chem. Soc.* **88**, 5511–5515.

[bb5] Bruker (2016). *APEX3* and *SAINT*. Bruker AXS Inc., Madison, Wisconsin, USA.

[bb6] Desiraju, G. R. & Steiner, T. (1999). *The Weak Hydrogen Bond in Structural Chemistry and Biology*, p49. Oxford University Press.

[bb7] Flack, H. D. & Bernardinelli, G. (1999). *Acta Cryst.* A**55**, 908–915. 10.1107/s0108767399004262 10927300

[bb8] Goren, M. B. & Kochansky, M. E. (1973). *J. Org. Chem.* **38**, 3510–3513. 10.1021/jo00960a016 4780822

[bb9] Groom, C. R., Bruno, I. J., Lightfoot, M. P. & Ward, S. C. (2016). *Acta Cryst.* B**72**, 171–179. 10.1107/S2052520616003954 PMC4822653 27048719

[bb10] Hooft, R. W. W., Straver, L. H. & Spek, A. L. (2008). *J. Appl. Cryst.* **41**, 96–103. 10.1107/S0021889807059870 PMC2467520 19461838

[bb11] Jerfy, A. & Roy, A. B. (1970). *Aust. J. Chem.* **23**, 847–852.

[bb12] Krause, L., Herbst-Irmer, R., Sheldrick, G. M. & Stalke, D. (2015). *J. Appl. Cryst.* **48**, 3–10. 10.1107/S1600576714022985 PMC4453166 26089746

[bb13] Li, C.-J., Lin, Z., Yun, L., Xie, Y.-L., Leng, J.-D., Ou, Y.-C. & Tong, M.-L. (2010). *CrystEngComm*, **12**, 425–433.

[bb14] Manteau, B., Genix, P., Brelot, L., Vors, J.-P., Pazenok, S., Giornal, F., Leuenberger, C. & Leroux, F. R. (2010). *Eur. J. Org. Chem.* pp. 6043–6066.

[bb15] Nantka-Namirski, P. & Rykowski, A. (1972*a*). *Acta Pol. Pharm.* **29**, 129–134 [Eng. Trans.]. 5082408

[bb16] Nantka-Namirski, P. & Rykowski, A. (1972*b*). *Acta Pol. Pharm.* **29**, 233–238. [Eng. Trans.]. 5082408

[bb17] Parkin, S. (2000). *Acta Cryst.* A**56**, 157–162. 10.1107/s010876739901497x 10772457

[bb18] Parkin, S. (2013). *CIFFIX*. https://xray.uky.edu/Resources/scripts/ciffix

[bb19] Parkin, S. & Hope, H. (1998*a*). *Acta Cryst.* B**54**, 339–344.

[bb20] Parkin, S. & Hope, H. (1998*b*). *J. Appl. Cryst.* **31**, 945–953.

[bb21] Parsons, S., Flack, H. D. & Wagner, T. (2013). *Acta Cryst.* B**69**, 249–259. 10.1107/S2052519213010014 PMC3661305 23719469

[bb22] Qiu, J., Zhang, Y., Yao, S., Ren, H., Qian, M., Hong, Q., Lu, Z. & He, J. (2019). *J. Bact.* **201**, e00665–18. 10.1128/JB.00665-18 PMC6416912 30692170

[bb23] Sheldrick, G. M. (2008). *Acta Cryst.* A**64**, 112–122. 10.1107/S0108767307043930 18156677

[bb24] Sheldrick, G. M. (2015*a*). *Acta Cryst.* A**71**, 3–8.

[bb25] Sheldrick, G. M. (2015*b*). *Acta Cryst.* C**71**, 3–8.

[bb26] Shukla, O. P. & Kaul, S. M. (1973). *Indian J. Biochem. Biophys.* **10**, 176–178. 4792924

[bb27] Shukla, O. P., Kaul, S. M. & Khanna, M. (1977). *Indian J. Biochem. Biophys.* **14**, 292–295. 612544

[bb28] Spek, A. L. (2020). *Acta Cryst.* E**76**, 1–11. 10.1107/S2056989019016244 PMC6944088 31921444

